# Development of Monoclonal Antibodies against HIV-1 p24 Protein and Its Application in Colloidal Gold Immunochromatographic Assay for HIV-1 Detection

**DOI:** 10.1155/2016/6743904

**Published:** 2016-03-16

**Authors:** Yi Ma, Chao Ni, Emmanuel E. Dzakah, Haiying Wang, Keren Kang, Shixing Tang, Jihua Wang, Jufang Wang

**Affiliations:** ^1^School of Bioscience and Bioengineering, South China University of Technology, Guangzhou 510006, China; ^2^National Engineering Laboratory of Rapid Diagnostic Tests, Guangzhou Wondfo Biotech Co., Ltd., Guangzhou 510663, China

## Abstract

Human immunodeficiency virus type 1 (HIV-1) p24 protein is the most abundant viral protein of HIV-1. This protein is secreted in blood serum at high levels during the early stages of HIV-1 infection, making it a biomarker for early diagnosis. In this study, a colloidal gold immunochromatographic assay (GICA) was established for detecting p24 protein using mouse monoclonal antibodies (mAbs). The HIV-1 p24 protein was expressed in* E. coli *strain BL21 and the purified protein was used to immunize mice. Stable hybridoma cell lines secreting anti-p24 monoclonal antibodies were obtained after ELISA screening and subcloning by limiting dilution. 34 different capture and labeling mAb pairs were selected by a novel antibody-capture indirect sandwich ELISA and then applied in GICA to detect p24 protein. The GICA method has a limit of detection (LOD) of 25 pg/mL and could detect p24 protein in all 10 positive samples obtained from the National Reference of HIV-1 p24 antigen. Out of 153 negative samples tested, 3 false positives results were obtained. The overall specificity of this test was 98.03%. The good sensitivity and specificity of this method make it a suitable alternative to provide a more convenient and efficient tool for early diagnosis of HIV infection.

## 1. Introduction

P24 protein is derived from the Gag protein of HIV-1 and plays an important role in viral core assembly and maturation [1, 2]. HIV-1 RNA, anti-HIV antibodies, and p24 antigen are viral markers which have been used as a target antigen for early detection of HIV-1 infection [3, 4]. Over the past two decades, HIV immunoassays have gone through first-generation (using viral lysate for IgG antibody detection), second-generation (using recombinant antigens for IgG antibody detection), third-generation (IgM and IgG antibodies detection), and fourth-generation (antibody and p24 antigen detection) immunoassay. These test kits have helped to shorten the “window period” and provide an early diagnosis for suspected HIV infected samples as compared with the third-generation immunoassays.

Enzyme linked immunosorbent assay (ELISA) is the most commonly used immunoassay in the third- and fourth-generation test kits for HIV diagnosis in China. The limit of detection (LOD) for p24 antigen ranges from 11 pg/mL to 70 pg/mL [5]. However, ELISA involves complicated procedures and requires long reaction time of at least 2 hours. GICA has therefore been recognized as a popular diagnostic tool for the detection of HIV antibody because of its user-friendly format and easy and rapid rate of obtaining results without the need for special equipment [6–8]. There is no comprehensive study on p24 antigen detection using GICA and thus, the development of HIV-p24 antigen GICA, which can be used simultaneously with the HIV antibody GICA for rapid HIV detection, is of great significance. In this work, mAbs were screened against recombinant p24 protein and its application in GICA for HIV-1 detection was explored.

## 2. Materials and Methods

### 2.1. Strains, Plasmids, Enzymes, and Reagents

Competent* E. coli *cells DH5*α* and BL21 (DE3) were purchased from TIANGEN Biotech (Beijing, China). Restriction endonucleases* Nde*I and* Xho*I, Premix LA Taq, and DNA Ligation Kit were purchased from TAKARA Biotechnology Co., Ltd. (Dalian, China). SP2/0-Ag14 myeloma cells and pET-28a plasmid were preserved in our lab. All chemicals used in this study were of analytical grade.

### 2.2. Samples Collection and Examination

The National Reference of HIV-1 p24 antigen containing 10 positive samples (P1 to P10) and 10 sensitivity samples (L1 to L10) was purchased from the National Institute for the Control of Pharmaceutical and Biological Products (Beijing, China). These sensitivity samples were p24 protein (WHO standards) from the National Institute for Biological Standards and Control (Hertfordshire, UK) and diluted to concentrations of L1 (20 IU/mL), L2 (10 IU/mL), L3 (5 IU/mL), L4 (2.5 IU/mL), and L10 (0 IU/mL). Serum samples (*n* = 153) were collected from the General Hospital of Guangzhou Military Command of PLA. HIV antibody and p24 negative samples were confirmed by two different commercially available fourth-generation HIV ELISA test reagents.

### 2.3. Gene Cloning and Recombinant Protein Expression

DNA sequence of the 149~354-amino acid sequence of HIV p55 Gag protein in the NCBI database (NP_057850.1) encoding the p24 gene was designed with codon optimization and synthesized by Shanghai Shenggong Co., Ltd. (Shanghai, China). The purified p24 PCR product and the pET28a(+) plasmid were both double digested with* Nde*I and* Xho*I restriction enzymes and ligated by T4 DNA ligase to construct pET28a-p24 plasmid.* E. coli* BL21 (DE3) with recombinant plasmid were cultured in LB medium supplemented with 50 *μ*g/mL kanamycin at 37°C until the logarithmic phase (at OD_600_ of 0.6~0.8) before induction with final concentration of 1.0 mM IPTG at 37°C, 25°C, and 18°C to OD_600_ of 2.0, respectively. Recombinant protein was purified on a HiTrap Ni^2+^ column using AKTAPurifier 100 (GE Healthcare Life Sciences, PA, USA).

### 2.4. Production of mAbs

Six-to-eight-week-old BALB/c mice were immunized subcutaneously with 100 *μ*g r-p24 mixed with the equal volume of Freund's complete adjuvant (Sigma-Aldrich, MN, USA). On the 14th and 28th day, mice were boosted with 50 *μ*g r-p24 mixed with the equal volume of Freund's incomplete adjuvant (Sigma-Aldrich, MN, USA). On day 38, a final injection of 50 *μ*g r-p24 in PBS was administered intraperitoneally. Hybridoma and mAbs were generated as described previously [9].

### 2.5. Antibody-Capture Indirect Sandwich ELISA

A novel antibody-capture indirect sandwich ELISA method was designed to screen the mAb pairs recognizing r-p24. ELISA microtiter plates were coated with different mAbs (10 *μ*g/mL in PBS, pH 9.6) and incubated overnight at 4°C followed by blocking with 3% BSA for 2 hr. For labeling of the detector antibody, 2 *μ*g/mL of each mAb was dissolved in PBS dilution buffer (containing 0.05% (v/v) Tween 20 and 1%  (v/v) BSA) and 0.1% (v/v) HRP conjugated goat-anti-mouse antibody (GAM-HRP) for 30 minutes before use. About 75 *μ*L r-p24 (1 *μ*g of protein/mL in PBS) containing 4% healthy mouse serum was incubated with 75 *μ*L detector mAb for 5 mins reaction time after which 100 *μ*L of the mixture was then added to each well in duplicate and then rinsed after 30 mins. The subsequent peroxidase reaction step was performed and all assay results were read with a microplate reader Thermo Scientific MK3 (Thermo Fisher Scientific, MA, USA) at wavelengths 450 nm.

### 2.6. Gold Immunochromatographic Assay for the Evaluation of p24 Protein

Monoclonal antibody pairs were used as capture and detector antibodies on the GICA platform. The detector antibody was labeled by conjugation to colloidal gold, mixed with active blocker against heterophilic antibodies and rheumatoid factor. The mixture was sprayed on glass fiber at 40 *μ*L/cm^2^. The capture mAb, goat-anti-mouse IgG, and active blocker were sprayed on a nitrocellulose membrane at a concentration of 2.0 mg/mL with a line thickness of 2 *μ*L/cm to form the test, control, and block lines, respectively. Immunochromatographic test strips were made as described previously [10]. A maximum of 70 *μ*L sample was added to the sample application site and the color development was observed after 20 mins.

### 2.7. GICA Test Strip Sensitivity to Recombinant p24 Antigen

Serial dilutions of r-p24 (1000 pg/mL, 250 pg/mL, 100 pg/mL, 50 pg/mL, 25 pg/mL, 10 pg/mL, and 0 pg/mL) were applied to the GICA test strips to check the sensitivity level at different antigen concentrations. Strips were observed for a maximum of 20 mins and the color development was compared with a standard color chart. The band intensity was then graded.

## 3. Results

### 3.1. Expression of Recombinant Protein

**Figure 1:**
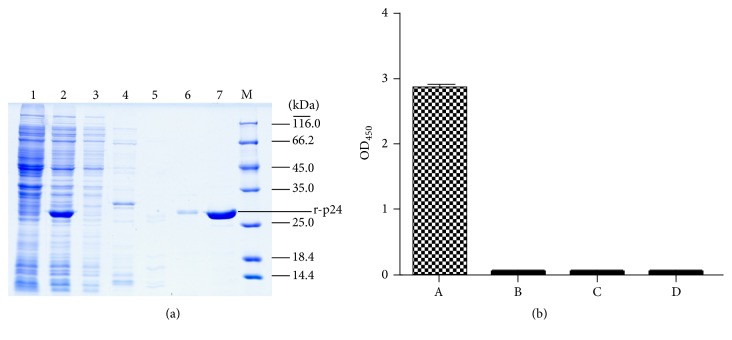
Preparation and identification of recombinant p24. (a) Expression and purification of recombinant p24. 1: whole cell lysate without IPTG induction, 2: whole cell lysate with IPTG induction, and 3: flow through solution; lanes 4 to 7 are four different solutions eluted by 50 mM, 150 mM, 300 mM, and 500 mM imidazole buffer, respectively. (b) Indirect ELISA analysis of the recombinant p24. A: antip24 antibody as the primary antibody, B: blank without primary antibody, C: blank without coating antigen, and D: blank without primary antibody and coating antigen.

It was observed that higher incubation temperatures resulted in the formation of inclusion bodies while lower incubation temperatures showed high expression levels of soluble r-p24 proteins. The expression was carried out at 18°C and the r-p24 protein was purified on a HiTrap Ni^2+^ column with a total purity of >95% (Figure 1(a), lane 7). The purified protein of interest was confirmed by ELISA analysis of the protein using commercially available monoclonal antibody against p24 antigen (Figure 1(b)).

### 3.2. Screening of mAbs

28 stable anti-r-p24 hybridoma clones were selected using indirect ELISA. Capture and detector mAb combinations were paired and selected from these 28 clones for reactivity against the immunogen through indirect sandwich ELISA. Of 784 (28 × 28) sandwich mAb combinations, 34 pairs could react with r-p24 at 1 *μ*g/mL and 13 of those showed strong sensitivity with the r-p24 protein. A total of 15 mAbs pairs could react with 1 *μ*g/mL r-p24 and only 2 mAbs pairs could react with 1 ng/mL r-p24. An antibody pair consisting of capture (3-1D5) and detector (4-8A10) showed a relatively higher sensitivity to p24 and were selected for later GICA tests.

### 3.3. Test Strip Sensitivity to Recombinant p24 Antigen

**Figure 2:**
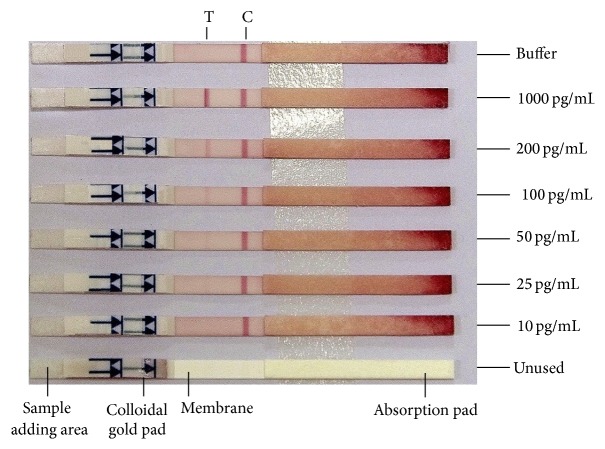
Sensitivity of GICA to r-p24 antigen. GICA test strips were treated with different concentrations (1000 pg/mL, 200 pg/mL, 100 pg/mL, 50 pg/mL, 25 pg/mL, and 10 pg/mL) of r-p24 antigen. T and C represent the test and control lines, respectively.

The sensitivity of the test strips using capture (3-1D5) and detector (4-8A10) was determined by treatment with different antigen concentrations (1000 pg/mL, 200 pg/mL, 100 pg/mL, 50 pg/mL, 25 pg/mL, 10 pg/mL, and 0 pg/mL) and the band intensity was evaluated after 20 mins by comparing with a standard color chart. Desired sensitivity for GICA was observed at 25 pg/mL of antigen (Figure 2).

### 3.4. GICA for HIV-1 p24 Positive and Negative Samples

GICA lateral strips made of 3-1D5 and 4-8A10 were used to detect HIV-1 p24 antigen positive serum samples from the National Reference (NR) of HIV and p24 negative serum samples (Table 1). P1 to P10 (p24 positive samples in the NR) were consequently detected by the GICA method. Of the p24 sensitivity samples in the NR (L1 to L10), the L1, L2, and L3 samples with higher concentrations of p24 protein were positively detected by GICA while the lower concentration samples (L4–L10) were negative. Compared with the fourth-generation ELISA test reagent Vironostika HIV Uni-Form II Ag/Ab (Biomérieux, Boxtel, The Netherlands), three false positives were observed in the GICA test when p24 negative serum samples (*n* = 153) were tested, giving an overall specificity of 98.03%.

## 4. Discussion

The number of new HIV infection cases has been on the increase despite numerous awareness programs and efforts to curtail the spread of infection. With the introduction of rapid HIV antibody test kits, HIV screening has become more decentralized with more tests done on an individual rather than batch [21]. Hence, there is an urgent need for simple, inexpensive, rapid, and accurate detection formats to shorten the window period of HIV testing and thereby reduce the risk of transmitting the virus from person to person during blood transfusion. GICA techniques have been applied in the third-generation HIV rapid detection reagents, but there are no commercialized available GICA rapid diagnostic reagents which can quickly detect HIV antibodies and HIV-1 p24 antigen in clinical samples. The development of rapid HIV testing reagents for p24 antigen detection could provide an easy detection method and would further enhance the sensitivity of currently available rapid test kits for early detection of HIV infection; hence, the production of HIV-1 p24 rapid GICA detection reagent is of great significance.

The GICA method described here could provide a much easier, rapid, and relatively inexpensive alternative to current ELISA protocols for p24 antigen detection. The LOD of 25 pg/mL for p24 is close to that observed in many commercially available antigen/antibody combination ELISAs. The GICA also demonstrated a good ability to detect low p24 concentrations in the National Reference of HIV-1 p24 antigen. This assay could detect all the 10 HIV-1 p24 positive serum samples including HIV-1 AE and B genotype samples. The sensitivity of the GICA was confirmed to be 5 IU/mL when WHO standard p24 sensitivity samples in the National Reference of HIV-1 p24 antigen were tested.

Chemiluminescent microparticle immunoassays and enzyme linked fluorescence assays have also been developed recently for commercial use, but these require complete packages of reagents and supports (Table 2). In the past decade, HIV-1 p24 antigen assays have significantly improved, as well as the introduction of new methodology such as nanoparticle-based biobarcode amplification assays, magnetic immunochromatography assays, and immunosensor assays (Table 2). It has also been reported that the ultrasensitive capacitive immunosensor assay can decrease the LOD of p24 antigen detection to about 7.9 × 10^−8 ^pg/mL (Table 2). However, these assays generally require complex instruments or well-trained technicians for their operation which ultimately limit their use in point-of-care testing, especially in remote areas of most developing countries.

In this research, a novel antibody-capture indirect sandwich ELISA method was used for the rapid screening of antibody pairs. The selected antibody pairs showed good performance when applied in both sandwich ELISA and GICA. A total of 28 mAbs were obtained for combination experiments to screen the mAb pairs that could function well on GICA platform. Among these pairs, only one antibody pair showed the expected sensitivity for use on the GICA platform. Nevertheless, it is anticipated that more sensitive antibody pairs could be obtained by optimizing the immunization and antibody preparation process so as to enhance the sensitivity and specificity of the GICA kits. Most GICA mAb pairs perform well on the ELISA platform and some other immunological assays such as chemiluminescence microparticle immunoassay and fluorescence labeled immunochromatography. GICA strips can be applied in qualitative detection or semiquantitative detection of antigens. These results showed that the mAb pair can detect 20 pg/mL p24 antigen in ELISA method with HRP system (data not shown). In addition, fluorescent secondary antibody instead of colloidal gold can be used [22], and the consequent signal can be detected accurately by machine for p24 quantification.

A rapid assay for the simultaneous detection of both p24 antigen and HIV specific antibody would provide a rapid diagnostic tool for screening HIV infected blood or serum specimen and may serve as a better substitute in commercially available fourth-generation ELISAs. Further researches could focus on improving the sensitivity and specificity of the GICA in detecting p24 antigen and HIV antibodies in a single device and thus accelerate their application in the fourth-generation HIV immunoassays.

## Figures and Tables

**Table 1:** The detection results of National Reference for HIV-1 p24 antigen by GICA strips.

Sample	Derivation of references	Genotype	Detection level
P1	P24 positive Sample 34#	AE	Faint
P2	P24 positive Sample 106#	B	Faint
P3	P24 positive Sample 116#	B	Medium
P4	P24 positive Sample 119#	B	Weak
P5	500 fold-diluted laboratory-grown virus sample A	Not given	Medium
P6	100 fold-diluted laboratory-grown virus sample A	Not given	Weak
P7	1000 fold-diluted laboratory-grown virus sample L	B	Faint
P8	10000 fold-diluted laboratory-grown virus sample L	B	Faint
P9	50 fold-diluted HIV-1 window period Sample P	AE	Faint
P10	WHO Standard p24 25 U/mL	B	Medium
L1	WHO Standard p24 20 U/mL	B	Medium
L2	WHO Standard p24 10 U/mL	B	Weak
L3	WHO Standard p24 5 U/mL	B	Faint
L4	WHO Standard p24 2.5 U/mL	B	Negative
L10	Buffer for L Series	B	Negative

Detection level: Band intensity was read independently by two individuals.

**Table 2:** Comparison of GICA with some published HIV-1 p24 assays in recent years.

Assay	Support	p24 detection limit	Execution time
Colloidal gold immunochromatographic assay (GICA) Commercial assays	Lateral flow test strip	25 pg/mL	20 min
Abbott ARCHITECT HIV Ag/Ab Combo (Chemiluminescentmicroparticle immunoassay)	ARCHITECT automated analyzer	18.4 pg/mL (SFTS standard)	26 min [11]
Roche Elecsys® HIV combi (Chemiluminescentmicroparticle immunoassay)	Modular automated analyzer	0.9 IU/mL (WHO standard)	18 min [12]
Biomérieux VIDAS HIV DUO Ultra (Enzyme linked fluorescence assay)	VIDAS automated analyzer	11.5 pg/mL (SFTS standard)	120 min [13]
Bio-Rad GS HIV Combo Ag/Ab (Enzyme linked immunosorbent assay)	Bio-Rad EVOLIS*™* Automated System	12.7 pg/mL (SFTS standard)	>120 min [14]
Bio-Rad Genscreen*™* ULTRA HIV Ag-Ab (Enzyme linked immunosorbent assay)	Microplate	13 pg/mL (SFTS standard)	120 min [13]
Laboratory assays			
Ultrasensitive capacitive immunosensor assay	Capacitive immunosensor system	7.9 × 10^−8^ pg/mL	20 min [15]
Amperometricimmunosensor assay based on direct gold electroplating-modified electrode	Amperometricimmunosensor system	8 pg/mL	20 min [16]
Amperometricimmunosensor assay based on acetone-extracted propolis	Amperometricimmunosensor system	6.4 pg/mL	10 min [17]
Boosted ELISA based on immune complex dissociation and amplified signal	Microplate	0.5 pg/mL	>120 min [18]
Nanoparticle-based biobarcode amplification assay	NanosphereVerigene ID Reader	0.1 pg/mL	>120 min [19]
Magnetic immuno-chromatography assay	MagnaBiosciences magnetic assay reader	30 pg/mL	40 min [20]

SFTS standard: French national reference of HIV-1 p24 Ag obtained from the French Society of Blood Transfusion.
